# Assessment of Functional and Pasting Properties of Fresh Orange Maize Hybrids and Open-Pollinated Varieties as Influenced by Genotype, Harvesting Time, and Growing Location

**DOI:** 10.3389/fnut.2021.757728

**Published:** 2021-11-24

**Authors:** Emmanuel Oladeji Alamu, Busie Maziya-Dixon, Abebe Menkir, Michael Adesokan, Olorunfemi Olaofe

**Affiliations:** ^1^Food and Nutrition Sciences Laboratory, Southern Africa Research and Administration Hub (SARAH) Campus, International Institute of Tropical Agriculture, Lusaka, Zambia; ^2^Food and Nutrition Sciences Laboratory, International Institute of Tropical Agriculture (IITA), Ibadan, Nigeria; ^3^Maize Breeding Unit, International Institute of Tropical Agriculture (IITA), Ibadan, Nigeria; ^4^Department of Chemistry, Ekiti State University, Ado Ekiti, Nigeria

**Keywords:** maize hybrid, open-pollinated varieties, maturity, genotypes, pasting properties, functional properties

## Abstract

The study evaluates the effects of genotype, maturity, and growing location on the functional and pasting properties of freshly harvested orange maize hybrids and open-pollinated varieties (OPVs). Eight fresh orange maize hybrid and eight fresh maize OPV, including the control, were harvested at three stages: 20, 27, and 34 days after planting (DAP). The freshly harvested maize samples were lyophilized and characterized for the pasting and functional properties using standard laboratory methods. The peak viscosity, final viscosity, and swelling power of the OPVs increased between 20 and 27 DAP. Additionally, the water absorption capacity increased between 20 and 27 DAP for the maize hybrids, with a decreasing trend between 27 and 34 DAP. However, genotypes 2, from the orange maize hybrid, and 5, amongst the OPV, were outstanding with the highest peak viscosities, indicating good final product quality. The combined ANOVA for the fresh orange maize hybrid and OPV showed a highly significant effect (*p* ≤ 0.01 and *p* ≤ 0.001) for the maturity and location on the pasting and functional properties except for the pasting temperature, final viscosity, and pasting time which showed no significant effect. In contrast, the location by genotypes by maturity interactions had no significant effects on the pasting and functional properties of the fresh maize hybrid and the orange OPV except only for the setback, which was highly significant at *p* ≤ 0.001. Nutritionists, food scientists, and maize breeders could use the information from this study to select the best maize genotypes at the appropriate harvesting period suitable for the production of the preferred maize-based products of consumers.

## Introduction

Maize (*Zea mays* L.) is a popular crop having a wide adaptability to different agroclimatic conditions. It is one of the most important annual cereal crops in the world (1). Maize is globally known as the queen of cereals because of its high yield potential amongst other cereal crops. It is cultivated on about 150,000,000 hectares in over 160 countries with a broader diversity of climate, soil, biodiversity, and management practices contributing to 36% of the global grain production. Maize is an important carbohydrate source for human diets in developing countries and animal feed in developed countries (2). The USA is the global leader in maize production, with 377,500,000 metric tons of maize (3) and 36% of the world total in 2014 (4). Maize can be successfully cultivated in a variety of soils ranging from loamy sand to clay loam. However, soils with good organic matter content and high water-holding capacity with neutral pH are considered suitable for higher productivity (5). Maize is processed and consumed in various forms, varying from region to region or from one ethnic group to another. For instance, maize grains are prepared by boiling, roasting, or as paste in Nigeria and Ghana, or as popcorn consumed all over West Africa (6). Maize provides about 1,400 Kcal/100 g of energy on a dry weight basis (7). Its common forms of consumption as an energy source for breakfast meals are cornflakes, tortillas, corn starch, tapioca, etc. In addition to being a source of carbohydrates, maize is a booster for fats, protein, and insoluble fibers, which helps in providing sufficient energy to meet the human daily dietary requirement (8).

The most cultivated maize are the white, yellow, and red maize varieties. Most people prefer the white and yellow varieties depending on the region. About 14 countries consume 85–95% of white maize as their staple food in sub-Saharan Africa. White maize also represents the leading staple food in Southern Africa, while most parts of South America and the Caribbean prefer yellow maize for animal feed (https://www.iita.org/cropsnew/maize). White maize, however, has limited micronutrients such as Vitamin A, Zinc, and Fe. The deficiencies of these essential micronutrients pose serious health concerns, especially in developing countries. Biofortification is a recent public health intervention that seeks to improve the micronutrient content of staple foods such as maize consumed by most poor people using conventional plant-breeding techniques to make a quantifiable impact on the extent of micronutrient malnutrition. Recently, plant breeders have developed biofortified varieties of maize that contain higher concentrations of beta-carotene, which are usually orange in color. Orange maize has been grown commercially in African countries like Nigeria, Ghana, and Zambia since 2013 (9).

Open-pollinated maize varieties are genetically diverse and developed by selecting open-pollinated corn ears that are desirable to the breeders or farmers. This causes a variation within plant populations that allow the varieties to adapt to local growing conditions and climates. Open-pollinated variety (OPV) seeds can be saved for future planting without losing vigor or yield (10). Hybrid varieties are the first generation of the cross that involves two or more inbred lines. They are genetically similar. Hybrid maize exhibits heterosis: the performance of the progeny of a cross between two pure-breeding lines superior to the parents. When a single-cross hybrid is allowed to open-pollinate, approximately half the hybrid vigor is lost. The crop produced from open-pollinated seeds harvested from a single-cross hybrid will not be as productive as the original single cross; therefore, seeds cannot be saved for the next planting (11).

Maize flour is frequently used in various pharmaceutical and food formulations globally. This flour is used either in its pure form or blended with other legumes such as groundnut and soybean (12). The industrial and food applications of this flour significantly depend on its functional characteristics. Functional properties such as water absorption, oil absorption, gelation, foaming, and emulsifying capacities are the inherent physicochemical properties that illustrate the structural behavior of food systems (13). The effect of harvesting time and processing method on the qualitative, physical, and chemical properties of OPV and hybrid maize has been previously reported (14–17). Mehrshad et al. (15) studied the effect of harvest time using moisture content (MC) to determine the best grain yield and reported that grains are best harvested when they have 20% MC. Maize yield increases with delayed harvesting time, but quality parameters such as crude fiber content decrease with the harvest period (17). Similar research by Panison et al. (18) on the effect of harvest time on the performance of maize hybrid reported that the delay in harvest time after physiological maturity increases the percentage of lodged and broken plants, reducing the actual grain yield. The authors also reported that the delay in harvest time does not affect grain mass in regions with high altitudes and latitudes. The most recent studies on maize are focused on the effects of harvesting time on its physicochemical properties (1, 14). However, similar studies on the effect of harvesting time on the pasting and functional properties of maize hybrid and OPV are scanty. Maize breeding efforts have improved agronomic traits, but the efforts have altered the functional and biophysical properties that determine the final processing and product quality (19). Considering the great importance of the functional properties of maize to its consumption pattern and in the food industry, it is also imperative to study the changes in the pasting properties of maize flour as affected by the period of harvesting and growing location. Thus, this study aimed to evaluate the effects of harvest time and growing location on the pasting and functional properties of OPVs and orange maize hybrids.

## Materials and Methods

### Genetic Material

Freshly harvested cobs from eight orange maize hybrids and OPVs were obtained from the experimental field of the International Institute of Tropical Agriculture (IITA) for the study. The viable seeds from these cobs were planted in two separate trials at Ibadan (7°22'N, 3°58'E, altitude 150 m) and Ikenne (10°40'N, 8°77'E, altitude 730 m) with different and known meteorological information, in the early seasons of April–August 2010 and 2011. The hybrids and OPV were arranged in a randomized complete block design (RCBD) with three replications. The cobs of each hybrid were self-pollinated to minimize contamination from other pollen sources. The description of the genetic materials, including the control used for the study, is provided in Supplementary Tables 1, 2.

### Field Sampling and Sample Preparations

Plants were pre-labeled randomly on the field at the three harvest maturity stages of 20, 27, and 34 days after pollination (DAP) for each hybrid (the day after pollination started from 50% anthesis or 50% silk emergence, 57 days after planting). They were harvested at 08:00 on the relevant dates. Twenty selected cobs of each hybrid were harvested from each plot and pooled to have 60 cobs/hybrid/harvest. A total of 192 samples were packed and taken to the laboratory as soon as possible (20). The samples were shelved and loaded into a glass beaker in the LABCONCO freeze dryer (South Kansas City, Missouri, USA) and freeze-dried for 72 h. The freeze-dried maize samples were pulverized into fine flour using a laboratory mill to a <0.8 micron particle size and then transferred into a Whirl park for further analysis.

### Laboratory Analysis

#### Determination of Functional Properties

##### Swelling Power and Solubility

The swelling power and solubility of the maize flour were determined by heating the flour-water slurry (0.35 g flour in 12.5 ml of distilled water) in a water bath at 60°C for 30 min, with constant stirring (21). The slurries were centrifuged for 15 min using a Super-speed centrifuge (Model No. L-708-2, Phillips Drucker, Oregon, USA) at 168 × g. The supernatant was decanted into a weighed evaporating dish and dried at 100°C for 20 min. The flour solubility was calculated by the difference in the weight of the evaporating dish. The swelling power was obtained by weighing the residue after centrifugation and dividing it by the original flour weight on a dry weight basis.

##### Water Absorption Capacity

The WAC was determined using the method described by Beuchat et al. (23). One gram of the samples was weighed into graduated 25 ml conical centrifuge tubes. Ten ml of distilled water was added and allowed to stand at 30 ± 2°C for 1 h. The suspension was centrifuged (Model No. L-708-2, Phillips Drucker, Oregon, USA) at 200 × g for 30 min. The supernatant was decanted, and the sample was reweighed. The weight change was expressed as the percent of the water absorption based on the original sample weight.

#### Determination of Pasting Properties

The pasting properties of the flour were determined in duplicate for each sample using a Rapid Visco Analyzer (RVA) (Model RVA-4; Newport Scientific Pty. Ltd., Warriewood, Australia). Three grams of the flour samples were weighed into a 25 ml canister, and 25 ml of distilled water was added. After thoroughly stirring the mixture, the canister was fitted into the RVA according to the instructions of the manufacturer. The slurry was heated from 50 to 95°C with a holding time of 2 min followed by cooling to 50°C with a 2 min holding time. The heating and cooling rate was constantly at 11.25°C/min. The pasting profile was determined with the aid of Thermocline for Windows Software (Newport Scientific Pty Ltd., Warriewood) connected to a computer.

#### Determination of the Degree of Starch Damage

The starch damage was determined using the standard method by the Association of Official Analytical Chemists (AOAC). The sample solution was made by dissolving 0.5 g of the flour samples with 20 mL of extractant. The flour samples were extracted for 15 min by shaking each flask for 10 s every 3 min at 30°C and filtered. Two milliliters of the filtrate were pipetted into a 25 ml volumetric flask containing 15 ml of distilled water at 21°C and 1 ml iodine solution. The mixture was left to stand for 10 min. The absorbance was 600 nm against a blank and was measured using a spectrophotometer (NOVA SPEC II, England). The starch damage was determined using the regression equation of Farrand (22). The analyses were in triplicate.

#### Statistical Analysis

The analysis of the results of the functional and pasting properties of the maize hybrid and OPV were at different stages of maturity were subjected to statistical analyses using the XLSTAT (Addinsoft, NY, USA) tools (24). An ANOVA was used to calculate the least squares means used to estimate the differences among the means of the pasting and functional properties for each genotype at 5% of the probability level. The mean, SD, coefficient of variation (CV), and SE values were also calculated.

## Results

Table 1 shows the mean squares (MS) from the ANOVA for the pasting and functional properties of the fresh orange maize hybrid and OPV maize evaluated at two growing locations. The combined ANOVA for the fresh orange maize hybrid and OPV showed a highly significant effect (*p* ≤ 0.01 and *p* ≤ 0.001) for maturity and location on the pasting and functional properties except for pasting temperature, final viscosity, and pasting time which showed no significant effect. Still, location and genotype had no significant effect (*p* > 0.05) on the pasting and functional properties except for the DSD of the fresh orange maize hybrid which showed a significant effect at *p* < 0.05. The effect of genotype and maturity was highly significant (*p* ≤ 0.001) on the solubility, peak time (*p* ≤ 0.01), pasting time, and DSD (*p* ≤ 0.05) of the fresh orange hybrid maize. Peak time and DSD had significant (*p* ≤ 0.001) genotype and maturity effects on the orange OPV. Location by genotypes by maturity interactions had no significant effects on the pasting and functional properties of the fresh maize hybrid and the orange OPV, except for the setback, which was highly significant at *p* ≤ 0.001.

**Table 1:** Mean squares from the ANOVA for the pasting and functional properties of the fresh orange maize hybrid.

**Parameters**	**DF**	**Peak 1**	**Trough 1**	**Break down**	**Final viscosity**	**Setback**	**Peak time**	**Pasting temp**	**DSD**	**Swelling power**	**Solubility**	**WAC**
Location	1	57.4	25.1	158^**^	2,280^**^	1,827^***^	0.96	4.75	350^***^	1.00	119	92,567^***^
Genotype	7	555^*^	233	75.3^**^	1,875^***^	846^*^	0.37	1,442	4.76	0.75	120^**^	2,853^***^
Maturity	2	3,260^***^	2,927^***^	133^**^	18,633^***^	6,815^***^	5.55^***^	1,689	41.8^*^	32.5^***^	284^**^	61,528^***^
Location * Genotype	7	246	141	22.	386	176	0.28	4.90	6.57^*^	0.53	79.1	327
Location *Maturity	2	1,203^**^	938^***^	69.3	490	2,405^***^	0.13	5.81	87.8^***^	15.80^***^	209^**^	31,381^***^
Genotype*Maturity	14	259	170	28.8	439	238^*^	1.00^**^	1,772^*^	18.1^*^	0.57	126^***^	461
Location * Genotype *Maturity	14	135	94.7	15.5	549	309^***^	0.46	4.39	10.6	0.60	100^**^	568
Error		220	135	25.8	443	209	0.44	838	9.92	0.43	43.80	664
**Mean squares from the ANOVA for the pasting and functional properties of fresh orange maize open-pollinated variety (OPV)**.												
**Parameters**	**DF**	**Peak 1**	**Trough 1**	**Breakdown**	**Final viscosity**	**Setback**	**Peak time**	**Pasting temp**	**DSD**	**Swelling power**	**Soluble**	**WAC**
Location	1	2,698	3,320	32.2^*^	1,515^*^	349^***^	0.96	4.75	180^**^	15.6^***^	266^***^	52,412^***^
Genotype	7	111^*^	97.5	6.23^**^	694^***^	389^*^	0.37	1,442	102^**^	0.33	14.0^*^	1,692^*^
Maturity	2	418^***^	380^***^	3.56^**^	18,951^***^	14,001^***^	5.55^***^	1,689	723^***^	30.1^***^	48.20^***^	25,314^***^
Location * Genotype	7	177	102	17.8	385	137	0.280	4.90	10.7	0.45	2.41	869
Location *Maturity	2	21.9^**^	168^***^	69.3	3,938	5,690^***^	0.130	5.81	10.7^**^	11.2^***^	55.0^***^	8,437^***^
Genotype *Maturity	14	175	142	14.4	512	210^*^	1.00^**^	1,772^*^	122^***^	0.31	8.46	702
Location * Genotype *Maturity	14	238	165	14.6	827	324^***^	0.46	4.39	5.86	0.59	5.84	651
Error		143	87.4	21.1	388	167	0.440	838	31.1	0.44	6.25	770

*, **, *** *Significant at P < = 0.05, P < = 0.01, and P < = 0.001 respectively; ns -Not significant P > 0.05; DSD, Degree of Starch Damaged; WAC, Water Absorption Capacity*.

*Data were from duplicate values, 2 field replications, 2 locations, and 3 maturity stages (N = 192)*.

The descriptive statistics for the fresh orange maize hybrid and orange OPV maize are presented in Tables 2, 3. The mean values of the peak viscosity, trough viscosity, breakdown viscosity, final viscosity, and swelling power increased between 20 and 27 DAP for the fresh orange maize hybrid. However, they decreased between 27 and 34 DAP. The reverse of this trend was observed for the mean values of the peak time, DSD, and WAC, where the values decreased between 20 and 27 DAP but increased between 27 and 34 DAP. Additionally, the mean values for setback viscosity increased and decreased significantly for solubility (*P* < 0.05) across the three maturity stages, whereas the mean value of the pasting temperature remained unaffected between 20 and 27 DAP but increased slightly at the last maturity stage. However, the mean values for peak viscosity, breakdown viscosity, peak time, final viscosity, setback viscosity, DSD, solubility, and WAC were significantly (*P* < 0.05) different between 20 and 27 DAP.

**Table 2:** Descriptive statistics of the pasting and functional properties of the fresh orange maize hybrid by maturity stages (*N* = 192).

**Maturity stages**		**Peak viscosity RVU**	**Trough viscosity RVU**	**Breakdown viscosity RVU**	**Final viscosity RVU**	**Setback viscosity RVU**	**Peak time min**	**Pasting temp ^**°**^C**	**DSD%**	**Swelling power**	**Solubility %**	**WAC %**
20DAP	^1^Mean	134b	103b	30.6b	191b	88.1b	5.70a	49.3b	4.19*a*	10.1b	9.21*a*	158a
	Min	105	86.0	18.6	142	56.1	5.49	49.2	2.67	9.31	7.44	150
	Max	150	116	41.2	218	111	5.80	49.4	5.53	11.0	13.31	167
	LSD (0.05)	13.3	11.6	5.75	21.6	19.5	0.163	0.064	0.570	0.658	1.53	9.00
	SE	1.88	1.27	0.886	2.98	2.30	0.014	0.006	0.135	0.070	0.277	0.632
	CV (%)	1.40	1.23	2.90	1.56	2.61	0.251	0.012	3.21	0.692	3.01	0.399
27DAP	Mean	156a	116a	40.0a	222a	106a	5.56b	49.3b	1.71*b*	11.2a	7.62*b*	123b
	Min	148.	108	34.3	195	74.0	5.44	49.3	1.15	10.8	6.45	117
	Max	173	128	44.5	238	130	5.66	49.4	2.43	11.9	9.98	130
	LSD (0.05)	13.3	11.6	5.75	21.6	19.5	0.163	0.064	0.570	0.658	1.53	9.00
	SE	0.947	0.825	0.405	2.04	2.28	0.010	0.006	0.045	0.049	0.154	0.454
	CV (%)	0.607	0.711	1.01	0.922	2.17	0.188	0.012	2.61	0.440	2.02	0.370
34DAP	Mean	138b	110ab	27.9b	219a	109a	5.83a	49.4a	2.21*b*	7.36c	7.18*b*	129b
	Min	126	100	17.8	201	86.5	5.70	49.3	1.93	6.96	6.20	119
	Max	151	119	31.9	244	136.0	6.08	49.4	2.48	8.21	8.55	160
	LSD (0.05)	13.3	11.6	5.75	21.6	19.5	0.163	0.064	0.570	0.658	1.53	9.00
	SE	0.908	0.762	0.571	1.96	1.90	0.014	0.006	0.023	0.051	0.093	1.64
	CV (%)	0.658	0.692	2.04	0.895	1.75	0.242	0.011	1.06	0.688	1.30	1.27

*Values with a similar letter in the column do not differ significantly (p < 0.05)*.

1 *Means were data from duplicate values, 2 replications, 2 locations, and 3 maturity stages (N = 192)*.

**Table 3:** Descriptive statistics of the pasting and functional properties of the fresh orange OPV maize by maturity stages (*N* = 192).

**Maturity**		**Peak viscosity RVU**	**Trough viscosity RVU**	**Breakdown viscosity RVU**	**Final viscosity RVU**	**Setback viscosity RVU**	**Peak time min**	**Pasting temp ^**°**^C**	**DSD%**	**Swelling power**	**Solubility %**	**WAC %**
20DAP	^1^Mean	160a	117a	43.8a	208b	91.8b	5.41b	49.3a	1.91b	10.8a	6.91b	128b
	Min	131	90.9	38.6	175	82.2	5.26	48.5	1.00	9.85	5.80	118
	Max	184	137	47.4	239	102	5.63	157	2.67	11.2	7.86	153
	LSD (0.05)	16.5	13.1	6.47	22.8	13.5	0.191	22.4	0.443	0.648	0.849	13.2
	SE	2.59	2.23	0.425	3.10	0.939	0.014	0.019	0.067	0.052	0.100	1.44
	CV (%)	1.62	1.91	0.972	1.49	1.02	0.250	0.039	3.51	0.485	1.44	1.12
27DAP	Mean	160a	118a	42.3a	223b	105b	5.61a	49.4a	2.32a	7.57b	8.23a	205a
	Min	151	107	33.7	200	92.3	5.28	49.3	1.77	6.91	7.11	178
	Max	175	128	49.2	253	127	5.87	49.7	2.77	8.62	9.41	235
	LSD (0.05)	16.5	13.1	6.47	22.8	13.5	0.191	22.4	0.443	0.648	0.849	13.2
	SE	0.898	1.00	0.726	2.10	1.57	0.022	0.000	0.036	0.067	0.092	2.05
	CV (%)	0.561	0.849	1.72	0.941	1.50	0.387	0.000	1.54	0.883	1.12	1.00
34DAP	Mean	148a	107a	40.9a	243a	135a	5.42b	49.3a	1.80b	6.94c	5.27c	125b
	Min	144	103	34.6	233	123	5.23	49.0	1.30	6.61	4.29	120
	Max	151.8	110	43.3	258	151	5.55	49.4	2.39	7.55	5.84	132
	LSD (0.05)	16.5	13.1	6.47	22.8	13.5	0.191	22.4	0.443	0.648	0.849	13.2
	SE	0.350	0.308	0.345	1.20	1.31	0.014	0.000	0.041	0.042	0.066	0.521
	CV (%)	0.236	0.287	0.842	0.493	0.969	0.256	0.000	2.28	0.605	1.25	0.418

*Values with a similar letter in the column do not differ significantly (p < 0.05)*.

1 *Means were data from duplicate values, 2 field replications, 2 locations, and 3 maturity stages (N = 192)*.

In contrast, the swelling power showed a mean value that is significantly different across the three maturity periods. Also, the pasting temperature shows no significant difference between 20 and 27 DAP, but there was a significant (*p* < 0.05) difference at 34 DAP. For the fresh orange OPV maize samples, at *p* > 0.05, the mean values of peak viscosity, trough viscosity, breakdown viscosity, and pasting temperature have no significant differences across the maturity periods. No significant difference exists between the mean values of the final viscosity and set back viscosity at maturity periods of 20 and 27 DAP but significantly different from 34 DAP. The swelling power and solubility have mean values significantly different from 20 to 27 to 34 DAP. In contrast, the peak time, DSD, and WAC have mean values that are not significantly different between 20 and 34 DAP.

Table 4 shows the pasting and functional properties of the fresh orange hybrid maize by genotypes and maturity stages. The results showed that the MC, trough, and pasting temperature for all the maize genotypes were not significantly different at 20, 27, and 34 DAP. In comparison, the WAC is significantly (*p* < 0.05) different at the different maturity periods for all the maize hybrid except for genotype 8, where the WAC at 27 and 34 DAP were not significantly different. The swelling power for genotypes 2, 3, 6, 7, and 8 are not significantly different, but genotypes 1 and 5 have significant differences (*p* < 0.05) in their swelling power at 20 and 27 DAP. The DSD differs significantly at 20, 27, and 34 DAP for genotypes 1, 2, 4, 6, 7, and 8, while solubility differs significantly at all the maturity stages for only genotypes 6 and 8. At all stages of maturity, the peak viscosity for genotype 4 had no significant difference, while other genotypes, including the control (genotype 8), had significant differences (*p* < 0.05) in their peak viscosity, mostly at 27 to 34 DAP. The final peak viscosity had no significant difference at 20, 27, and 34 DAP for all the maize hybrid except for genotypes 2 and 8. Also, genotypes 2, 5, and 6 had no significant difference in their setback viscosity.

**Table 4:** The pasting and functional properties of the fresh orange maize hybrid by genotypes and maturity stages (*N* = 192).

**Genotype**	**Maturity stage**	**MC**	**Swelling power**	**Soluble**	**WAC**	**DSD**	**Peak 1**	**Trough 1**	**Breakdown**	**Final viscosity**	**Setback**	**Peak time**	**Pasting temperature**
		**%**		**%**	**%**	**%**	**RVU**	**RVU**	**RVU**	**RVU**	**RVU**	**min**	**^**°**^C**
1	20D	8.67a	10.02abcd	7.93bc	156.64abcdef	3.06def	149.63ab	116.09a	33.54abc	218.06a	101.98ab	5.77ab	49.39a
1	27D	9.39a	11.57ab	6.59c	129.62bcdefghi	1.15g	150.59ab	108.40a	42.19a	238.07a	129.67a	5.44b	49.35a
1	34D	7.48a	11.04ab	7.35bc	121.81hi	1.81fg	158.67a	116.04a	42.63a	233.40a	117.35ab	5.44b	49.35a
2	20D	8.35a	10.11abc	7.44bc	149.95abcdefgh	5.17ab	150.29ab	109.09a	41.21a	207.61ab	98.52ab	5.65ab	49.34a
2	27D	7.61a	7.42e	6.93c	159.83abc	2.17efg	142.38ab	112.40a	29.98abc	233.44a	121.04ab	5.76ab	49.41a
2	34D	6.87a	10.98ab	6.45c	122.03hi	1.59fg	172.75a	128.21a	44.54a	237.96a	109.75ab	5.54ab	49.39a
3	20D	7.83a	7.03e	7.86bc	127.45cdefghi	2.23efg	139.88ab	108.09a	31.80abc	244.13a	136.04a	5.70ab	49.44a
3	27D	7.05a	8.21cde	6.20c	124.41fghi	2.00fg	150.50ab	118.56a	31.94abc	230.65a	112.09ab	5.80ab	49.42a
3	34D	9.28a	11.89a	7.21c	121.25hi	1.60fg	156.73a	117.02a	39.71a	217.21a	100.19ab	5.64ab	49.28a
4	20D	8.36a	10.42abc	8.59abc	154.91abcdefg	5.20ab	144.67ab	111.50a	33.17abc	195.73ab	84.23ab	5.75ab	49.28a
4	27D	8.43a	10.55abc	8.19bc	161.17ab	5.53a	129.73ab	92.71a	37.02abc	203.56ab	110.86ab	5.49ab	49.32a
4	34D	9.59a	10.17abc	8.11bc	158.74abcd	4.56abc	130.94ab	103.08a	27.85abc	203.65ab	100.56ab	5.79ab	49.27a
5	20D	6.23a	10.84ab	9.98abc	116.46i	1.61fg	154.38a	115.67a	38.71a	219.90a	104.23ab	5.62ab	49.30a
5	27D	6.97a	10.96ab	7.57bc	123.85ghi	1.82fg	148.15ab	107.65a	40.50a	229.02a	121.37ab	5.53ab	49.30a
5	34D	7.26a	7.12e	7.39bc	125.29efghi	2.30efg	140.44ab	111.34a	29.11abc	219.52a	108.19ab	5.85ab	49.34a
6	20D	7.01a	10.91ab	8.96abc	121.99hi	2.43defg	155.13a	120.86a	34.27abc	194.90ab	74.04ab	5.66ab	49.26a
6	27D	8.50a	9.37bcde	12.13ab	159.96abc	3.84bcd	135.04ab	108.65a	26.40abc	179.29ab	70.65ab	5.76ab	49.27a
6	34D	7.72a	7.19e	7.07c	126.67defghi	2.39defg	134.90ab	117.11a	17.79c	203.60ab	86.50ab	6.08a	49.37a
7	20D	7.66a	7.33e	6.85c	130.73bcdefghi	2.48defg	125.92ab	99.96a	25.96abc	208.82ab	108.86ab	5.77ab	49.44a
7	27D	7.30a	11.55ab	6.89c	123.38ghi	1.68fg	151.60ab	114.15a	37.46ab	201.63ab	87.48ab	5.57ab	49.27a
7	34D	9.04a	9.31bcde	13.31a	167.35a	2.67def	125.63ab	98.71a	26.92abc	180.42ab	81.71ab	5.57ab	49.29a
8	20D	8.62a	10.95ab	7.96bc	157.02abcde	3.50cde	104.65b	86.04a	18.60bc	142.13b	56.08b	5.80ab	49.24a
8	27D	7.34a	7.61de	6.59c	118.51hi	1.93fg	138.02ab	108.54a	29.48abc	210.69ab	102.15ab	5.79ab	49.33a
8	34D	7.13a	6.96e	8.55abc	118.96hi	2.17efg	132.62ab	105.25a	27.38abc	200.75ab	95.50ab	5.85ab	49.34a

*Values with similar letters in the column do not differ significantly (p < 0.05)*.

*MC, Moisture content; WAC, Water Absorption Capacity; DSD, Degree of Starch Damaged; RVU, Rapid Visco Unit*.

Table 5 shows the pasting and functional properties of the fresh orange OPV maize by genotypes and maturity stages. The pasting properties, including peak viscosity, trough, breakdown, final viscosity, peak time, and pasting temperature for genotypes 1 to 8 had no significant differences at 20, 27, and 34 DAP except for the setback viscosity, which was significantly different (*p* < 0.05) across the different stages of maturity. Also, for the functional properties, the swelling power and solubility for all the OPV genotypes were significantly different at 20, 27, and 34 DAP, although the solubility of genotype 2 had no significant difference between 20 and 27 DAP. In contrast, genotype 8 shows no significant difference at 20 and 34 DAP, respectively. The setback of genotypes 3, 7, and 6 shows a significant (*p* < 0.05) difference at all the stages of maturity. In comparison, genotypes 5 and 2 had no significant (*P* > 0.05) difference in setback viscosity between 20 and 27 DAP, and no significant difference in the setback viscosity for genotypes 1 and 4 between 27 and 34 DAP.

**Table 5:** The pasting and functional properties of the unprocessed fresh orange OPV maize by genotypes and maturity stages (*N* = 192).

**Genotype**	**Maturity stage**	**MC**	**Swelling power**	**Soluble**	**WAC**	**DSD**	**Peak 1**	**Trough 1**	**Breakdown**	**Final viscosity**	**Setback**	**Peak time**	**Pasting temperature**
		**%**		**%**	**%**	**%**	**RVU**	**RVU**	**RVU**	**RVU**	**RVU**	**min**	**^**°**^C**
1	20D	8.08a	9.85abc	7.51abcde	117.48e	2.44ab	131.42a	91.00a	40.42a	176.44a	85.44de	5.26a	49.48a
1	27D	6.15a	7.94bcd	7.92abcd	208.57ab	2.49ab	159.00a	123.63a	35.38a	224.09a	100.46abcde	5.77a	49.69a
1	34D	7.33a	6.66d	4.84ef	131.96de	1.62ab	150.02a	108.02a	42.00a	240.46a	132.44abcde	5.23a	49.13a
2	20D	5.83a	10.97a	7.82abcd	126.05e	2.67a	173.69a	126.46a	47.23a	220.13a	93.67bcde	5.38a	48.92a
2	27D	7.08a	7.43cd	7.91abcd	199.44abc	2.18ab	162.21a	119.54a	42.67a	214.96a	95.42bcde	5.64a	49.39a
2	34D	6.70a	7.27cd	5.78cdef	121.94e	2.00ab	150.52a	109.96a	40.57a	232.75a	122.79abcde	5.53a	49.30a
3	20D	6.20a	10.96a	6.61abcdef	119.47e	1.63ab	163.73a	119.44a	44.29a	210.00a	90.57de	5.40a	49.28a
3	27D	6.79a	8.62abcd	7.85abcd	235.19a	2.28ab	154.67a	115.46a	39.21a	211.42a	95.96bcde	5.64a	49.44a
3	34D	6.65a	6.86d	4.95def	122.24e	1.94ab	151.79a	108.46a	43.34a	252.15a	143.69abc	5.38a	49.03a
4	20D	7.81a	10.53ab	6.85abcdef	153.01cde	2.20ab	131.79a	90.88a	40.92a	174.75a	83.88de	5.63a	49.44a
4	27D	7.32a	7.23cd	8.76ab	200.51ab	2.33ab	156.42a	107.25a	49.17a	212.96a	105.71abcde	5.28a	49.34a
4	34D	7.53a	6.93d	5.45cdef	129.54e	1.82ab	143.83a	109.21a	34.63a	234.35a	125.15abcde	5.55a	49.29a
5	20D	7.48a	11.03a	5.80bcdef	118.99e	1.75ab	184.27a	136.90a	47.38a	239.11a	102.21abcde	5.39a	48.64a
5	27D	7.29a	7.25cd	7.11abcdef	202.34ab	2.28ab	161.75a	128.08a	33.67a	230.42a	102.33abcde	5.87a	49.34a
5	34D	7.14a	6.62d	4.29f	123.07e	2.39ab	149.42a	107.15a	42.27a	258.23a	151.08a	5.45a	49.35a
6	20D	7.07a	10.89a	7.86abcd	130.09e	1.00b	174.04a	127.36a	46.69a	225.69a	98.33bcde	5.34a	49.43a
6	27D	6.25a	6.91d	9.41a	215.06ab	2.47ab	151.13a	107.42a	43.71a	199.75a	92.34cde	5.54a	49.33a
6	34D	7.77a	7.05cd	5.52cdef	125.78e	1.54ab	149.83a	109.15a	40.69a	235.29a	126.15abcde	5.43a	49.25a
7	20D	8.02a	10.75a	6.95abcdef	130.05e	1.55ab	176.11a	131.44a	44.67a	229.79a	98.35bcde	5.40a	48.52a
7	27D	6.19a	7.39cd	7.96abc	177.80bcd	2.77a	159.79a	114.55a	45.25a	235.50a	120.96abcde	5.61a	49.38a
7	34D	7.63a	6.61d	5.47cdef	122.74e	1.82ab	146.29a	105.32a	40.98a	250.54a	145.23ab	5.48a	49.34a
8	20D	6.67a	11.16a	5.89bcdef	132.80de	2.03ab	147.44a	108.88a	38.56a	191.04a	82.16e	5.48a	48.52a
8	27D	6.83a	7.81bcd	8.91a	197.60abc	1.77ab	175.25a	126.17a	49.09a	253.25a	127.09abcde	5.54a	49.39a
8	34D	7.08a	7.55cd	5.84bcdef	119.77e	1.30ab	145.60a	102.52a	43.09a	238.02a	135.50abcd	5.31a	49.36a

*Values with similar letters in the column do not differ significantly (p < 0.05)*.

*MC, Moisture content; WAC, Water Absorption Capacity; DSD, Degree of Starch Damaged; RVU, Rapid Visco Unit*.

**Figure 1:**
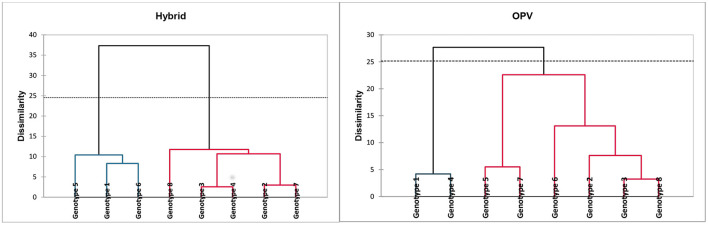
Dendrograms of hybrid and OPV orange maize genotypes.

Tables 6, 7 show the cluster analysis of the fresh maize hybrid and OPV using the pasting and functional properties. Genotypes 1 to 7, including genotype 8, which was used as control, were clustered into groups using their pasting and functional properties for the OPV and maize hybrid separately. Figure 1 shows the dendrograms for the orange OPV and maize hybrid. For the fresh orange maize hybrid, genotypes 1, 5, and 6 belong to cluster 1, while genotypes 2, 3, 4, 7, and 8 (control) were grouped into cluster 2. The pasting and functional properties in cluster 1 were slightly higher than in cluster 2, except for the DSD and solubility in cluster 2, which were slightly higher than those in cluster 1. Also, for the orange OPV genotypes, cluster 1 comprises genotypes 1 and 4, while genotypes 2, 3, 5, 6, 7, and 8 were contained in cluster 2 (Table 7). These genotypes have similarities in their functional and pasting properties with the control, making them a choice for selection over genotypes 1 and 4.

**Table 6:** Clusters of fresh orange hybrid and OPV using all parameters.

**Hybrid**		**OPV**	
**Cluster 1**	**Cluster 2**	**Cluster 1**	**Cluster 2**
Genotype 1	Genotype 2	Genotype 1	Genotype 2
Genotype 5	Genotype 3	Genotype 4	Genotype 3
Genotype 6	Genotype 4		Genotype 5
	Genotype 7		Genotype 6
	Genotype 8		Genotype 7
			Genotype 8

**Table 7:** Cluster centroids for hybrids and OPVs functional and pasting properties.

**Cluster**	**Swelling power**	**Soluble**	**WAC**	**DSD**	**Peak 1**	**Trough 1**	**Breakdown**	**Final viscosity**	**Setback**	**Peak time**	**Pasting temp**
**Hybrid**											
Cluster 1	9.71	7.19	138.88	2.61	149.30	113.44	35.86	225.64	112.20	5.68	49.36
Cluster 2	9.47	8.49	135.20	2.76	138.63	107.61	31.03	201.56	93.95	5.70	49.31
**OPV**											
Cluster 1	8.19	6.89	156.85	2.15	145.41	105.00	40.42	210.51	105.51	5.45	49.39
Cluster 2	8.51	6.77	151.13	1.97	159.86	116.90	42.96	229.33	112.43	5.49	55.18

## Discussion

### Pasting Properties

The maturity stages affected the pasting and functional properties of the fresh orange OPV and maize hybrid. Most of the functional properties of carbohydrate foods are explained based on the structure and amount of starch and protein in the different cultivars (25, 26). The peak viscosity of the fresh orange maize hybrid 3, 5, 6, and 7 at 20 DAP was higher than the grand mean value of 134 rapid Visco units (RVU), while varieties 5 and 6 had the highest value of 150 RVU for peak viscosity. However, the peak viscosity of varieties 1, 6, and 8 increased above the grand mean of 138 RVU. Also, at 34 DAP, varieties 1, 5, 6, and 7 showed a higher peak viscosity than the grand mean value of 138 RVU, but variety 6 had the highest, having 151 RVU. The peak viscosity in this study is higher than the range of 57.9–114.9 RVU reported by Abdulazeez et al. (27). The peak viscosity indicates the strength of the pastes formed from gelatinization during processing in food applications. The pasting properties of starch are affected by the amylose content, amylose/amylopectin ratio, lipids, and protein content (28). The starch concentration also influences the pasting properties in a solution, and high starch content requires a shorter time and low temperature to swell, thereby increasing the peak viscosity compared with solutions with very low starch content (29).

The study shows that variety 6 showed high peak viscosity at all stages of maturity while varieties 5 and 7 showed higher peak viscosity at 20 and 34 DAP. An increase in the peak viscosity could be attributed to an increased water absorption rate and swollen starch granules during heating (30). Additionally, for the fresh maize hybrid, it was observed that at 20 DAP, varieties 2, 3, 5, 6, and 7 had a higher peak viscosity than the grand mean of 160 RVU, while variety 5 had the highest value of 184 RVU. At 27 DAP, a higher peak viscosity than the grand mean of 160 RVU was observed for varieties 2, 5, 7, and 8. The differences in the pasting properties of the various genotypes might be related to the starch composition and structures (31). Also, high peak viscosity could be due to low protein and lipid content. During pasting, the swelling of the starch increases due to low protein and lipid content, which might increase its viscosity (32).

Meanwhile, at 34 DAP, varieties 1, 2, 3, 5, and 6 showed higher peak viscosity than the grand mean of 148 RVU, and variety 5 had the highest value of 152 RVU. These values were lower than the findings of Adedeji and Tadawus (33), where the average peak viscosity of maize hybrid was reported to be 473.83 RVU. Peak viscosity has also been reported to be associated closely with the degree of starch damage, and high starch damage results in high peak viscosity (34). Also, the mean values for setback viscosity increase across the three maturity stages, whereas the mean value of the pasting temperature remained unaffected between 20 and 27 DAP but increased slightly at the third maturity stage. The pasting temperature measures the minimum temperature required to cook foodstuff (35). The low pasting temperature of the maize flours suggests that they quickly formed pastes, hence, more suitable in most food and non-food industrial processes. A high pasting temperature indicates a higher resistance toward the swelling of the maize starch granules (36). The setback viscosity of the fresh orange hybrid maize at 20 DAP was 88.10 and at 34 DAP 109 RVU, while the OPV was at 91.80 and 135 RVU. The setback viscosity of OPV and hybrid increases as the maize matures. The setback is referred to as the ability to retrograde, which indicates the re-association of the amylose molecules released during gelatinization (37). Onabanjo et al. (38) reported a setback viscosity ranging from 60.70–74.80 RVU for the maize wheat blend. This range is lower than the values observed in this current study for OPV and maize hybrid. Variations in the paste properties of the starches of the different maize genotypes are essential in producing commercially resistant starches (39), among other uses.

### Functional Properties

The functional properties of the orange OPV maize genotypes and maize hybrid at different stages of maturity are presented in Tables 3, 4, respectively. The WAC was affected by maturity stages for the maize hybrid, including genotype 8 (control). The differences in WAC amongst the genotypes and maturity stages could result from many factors, including the protein (40) and crude fiber content (41). The degree of starch damage could also contribute to changes in the WAC. This is evident in the significant difference in the DSD for all the maize hybrids at all stages of maturity. However, the DSD for the orange OPV was not affected by the maturity stages for all the genotypes, including the control. The maize genotype 7 had the highest WAC (167.35%) at 34 DAP, higher than genotype 8 (control) that had 118.96% at 34 DAP for the maize hybrid. The WAC for the OPV ranged from 117% for genotype 7 at 27 DAP to 235% for genotype 3 at 27 DAP, which is in close agreement with the 173–235% reported by Oladapo et al. (42) for white and yellow maize flour, respectively. The swelling power for genotypes 1 and 5 is significantly different at 20 and 27 DAP, while genotypes 2, 3, 4, 6, 7, and control had no significant changes in swelling power across the maturity periods. Genotype 3 at 34 DAP had the highest swelling power of 11.89%, which is different significantly from the control (6.96%) at 34 DAP and higher than the value of 6.13% reported for yellow maize flour (42). However, a similar trend was established as swelling power mainly increases with the period of maturity. Swelling power reflects the hydration capacity of the insoluble fraction of the maize starch. The higher the swelling power, the greater the digestibility and utilization of the flour for dietary applications (33, 43, 44). Genotypes 2 and 3 had 7.44 and 7.86% solubilities, respectively, which have similarities with the control (genotype 8) with a solubility of 7.96 at 20 DAP as there were no significant differences in the solubilities. The solubility index for all maize hybrids in the present study is higher than 5.28% reported by Makanjuola and Makanjuola (45) for yellow maize starch. In this study, genotype 5 had the highest solubility of 12.13% at 27 DAP, which is consistent with the findings of Alamu et al. (46) for a solubility index of 12.69% in 100% maize flour.

### Location, Maturity, and Genotypes Interactions on Pasting and Functional Properties

The combined ANOVA for the pasting and functional properties of the fresh orange hybrid and OPV maize (Table 1) shows that location and maturity significantly impact all the pasting properties except for breakdown viscosity, final viscosity, and pasting time. There was no significant location × genotype interaction MS on the pasting and functional properties for the orange OPV. However, DSD showed significant location × genotypes interaction for the orange maize hybrid. Also, setback viscosity showed significant location × genotype × maturity interactions for both the OPV and orange maize hybrid. Alamu et al. (14) also reported significant location × maturity interactions on specific physical properties of maize hybrids.

Similarly, the peak time and DSD of the orange OPV had significant genotype and maturity interactions. The present study results revealed that location and genotype interaction did not significantly affect the pasting and functional properties of the maize hybrid and OPV. However, most of the properties were affected chiefly by the interaction of location and stages of maturity.

## Conclusion

The present study has shown that the stages of maturity affect the pasting and functional properties of the orange OPV and maize hybrids. Higher peak viscosity was observed with maize hybrids 2, 3, 5, and 6. Variety 2 had an outstanding peak viscosity at all stages of maturity, though the highest peak viscosity for genotype 2 was at the 34 DAP maturity stage. This makes maize hybrid 2 the best choice based on its peak viscosity, indicating the quality of the final products. Cluster analysis also indicates that genotype 2 had similarities with genotype 8 (control). Also, genotypes 2, 5, and 7 are best amongst the open-pollinated genotypes with high peak viscosity. Genotype 5 was most preferred, with the highest peak viscosity at 20 DAP. Similarly, genotype 5 was grouped with the control in the cluster analysis for the orange OPV maize. The OPV varieties had higher setback viscosity than the maize hybrid; this indicates an unlikely tendency for maize hybrid to retrograde and better product stability.

## Data Availability Statement

The raw data supporting the conclusions of this article will be made available by the authors, without undue reservation.

## Author Contributions

EA and BM-D designed the study. EA and AM conducted the experiments. EA and MA analyzed and interpreted the datasets and prepared the manuscript. OO, BM-D, and AM supervised the study and edited the manuscript. All authors contributed to the article and approved the submitted version.

## Funding

This research was supported by the International Institute of Tropical Agriculture (IITA) Ibadan, Nigeria, and the Bill & Melinda Gates Foundation (BMGF) through a grant OPP1178942. This work was also supported by Crop (CRP) Maize under Consultative Group on International Agricultural Research (CGIAR), a global partnership for a food-secure future.

## Conflict of Interest

The authors declare that the research was conducted in the absence of any commercial or financial relationships that could be construed as a potential conflict of interest.

## Publisher's Note

All claims expressed in this article are solely those of the authors and do not necessarily represent those of their affiliated organizations, or those of the publisher, the editors and the reviewers. Any product that may be evaluated in this article, or claim that may be made by its manufacturer, is not guaranteed or endorsed by the publisher.
