# Supplementary material for: Assessment of Functional and Pasting Properties of Fresh Orange Maize Hybrids and Open-Pollinated Varieties as Influenced by Genotype, Harvesting Time, and Growing Location
Source: Front Nutr. 2021 Nov 24;8:757728. doi: 10.3389/fnut.2021.757728 (PMC8653809; doi:10.3389/fnut.2021.757728)
Supplement: Supplementary file 1 [file Table_1.docx]

**Supplementary Table 1: Genotype name of selected yellow hybrid maize trial**

| **s/n** | **Pedigree** | **Source** |
| --- | --- | --- |
| 1 | ACR97TZL-CCOMP1-Y-S3-13-1-B-B-B-B-B-B-B/9450xKI 21-3-2-2-1-3-B-B-B-B-B-B-B-B-B | 09C8073B |
| 2 | (GT-MAS:Gk x BABANGOYO x GT-MAS:Gk)-2-1-3-1-B-B-B-B-B-B-B-B-B-B/(MP420 x 4001 x MP420)-3-1-2-1-B-B-B- | 09C8075B |
| 3 | (KU1409/KU1414-SR/KVI43)-S2-4-1-  BB/4001xB73LPAx4001-33-2-1-B*4 | 09C8087B |
| 4 | (KU1409/KU1414-SR/NC298)-S2-8-1-BB/9450xKI21-1-5-3 2-2-B*5 | 09C8089B |
| 5 | (KU1409/KU1414-SR/NC298)-S2-7-1-BB/9450xKI21-7-3-1-2-4-B*4 | 09C8095B |
| 6 | (KU1409/KU1414-SR/KUI2007)-S2-3-2-BB/9450xKI21-1-5-2-1-2-B*5 | 09C8097B |
| 7 | 9450xKI21-7-2-1-2-B*4/KU1409xMO17LPAxKU1409-27-3-1-1-B*7 | 09C8099B |
| 8 | Oba Super-II ( Control) |  |
