# Supplementary material for: Assessment of Functional and Pasting Properties of Fresh Orange Maize Hybrids and Open-Pollinated Varieties as Influenced by Genotype, Harvesting Time, and Growing Location
Source: Front Nutr. 2021 Nov 24;8:757728. doi: 10.3389/fnut.2021.757728 (PMC8653809; doi:10.3389/fnut.2021.757728)
Supplement: Supplementary file 2 [file Table_2.docx]

**Supplementary Table 2: Genotype name of selected yellow open-pollinated varieties (OPV) maize trial**

| Entry | Name | Source | Origin | Grain colour |
| --- | --- | --- | --- | --- |
| 1 | PVA SYN3 F2 | 07A04139 | IITA | Yellow |
| 2 | PVA SYN2 F2 | 09A2541 | IITA | Yellow |
| 3 | PVA SYN6 F2 | 09A2543 | IITA | Yellow |
| 4 | PVA SYN8 F2 | 09A2545 | IITA | Yellow |
| 5 | PVA SYN10 F2 | 09A2547 | IITA | Yellow |
| 6 | TZLCOMP1 SYN-Y-1 | - | IITA | Yellow |
| 7 | POP66SR/ACR91SUWAN1-SRC1/ACR91SUWAN1-SRC1 | 05C04830 | IITA | Yellow |
| 8 | Acr. 91 Suwan 1-SR C1 (Control) | - | IITA | Yellow |
